# Survival benefit of resin cartridge extracorporeal blood purification therapy in patients with septic shock

**DOI:** 10.55730/1300-0144.5773

**Published:** 2023-11-11

**Authors:** Serdar EFE, Pervin HANCI, Volkan İNAL

**Affiliations:** 1 Division of Intensive Care, Department of Internal Medicine, Faculty of Medicine, Trakya University, Edirne, Turkiye; 2 Division of Intensive Care, Department of Pulmonary Medicine, Faculty of Medicine, Trakya University, Edirne, Turkiye

**Keywords:** Blood purification, critical care, extracorporeal therapy, sepsis, septic shock

## Abstract

**Background/aim:**

Extracorporeal blood purification (EBP) therapies have shown promise as potential rescue treatments for patients with septic shock. However, precise evidence regarding their effectiveness is lacking. This case-control study aimed to evaluate the 28-day survival benefit of a resin cartridge-based EBP therapy compared to conventional therapies in patients with septic shock.

**Materials and methods:**

The study sample was collected retrospectively from the medical records of patients admitted to the intensive care unit (ICU) between 2015 and 2020. The study included patients with septic shock aged ≥18 years who had ICU stays >96 h and excluded those lost to follow-up by 28 days or readmitted. First, 28-day survival was compared between EBP patients and 1:1 matched conventionally treated controls. Second, the EBP patients were evaluated for clinical and laboratory improvements within 72 h of EBP therapy.

**Results:**

Of 3742 patients, 391 were included in this study, of whom 129 received EBP therapy and had a 28-day survival rate of 44%, compared to 262 matched controls who received conventional therapy alone and had a survival rate of 33% (p = 0.001, log-rank = 0.05, number needed to treat = 8, and odds ratio = 1.7). After receiving EBP therapy for 72 h, improvements were observed in the Sequential Organ Failure Assessment scores (p < 0.05), shock indices (p < 0.05), partial pressure of oxygen in the arterial blood to the fraction of inspiratory oxygen concentration ratios (p < 0.001), vasopressor requirements (p < 0.001), pH (p < 0.05), lactate levels (p < 0.001), and C-reactive protein levels (p < 0.05).

**Conclusion:**

The findings suggest that administering resin cartridge-based EBP therapy to patients with septic shock may improve their survival compared to conventional therapies.

## 1. Introduction

Sepsis is a life-threatening organ dysfunction caused by dysregulated host response to infection [1]. Despite advances in diagnosis and management, sepsis and septic shock remain significant global health concerns with high incidence and mortality rates [1, 2]. Therefore, clinical and experimental studies continue to explore adjunctive therapies [3]. Organ failure in sepsis has been attributed to a dysregulated host response characterized by the release of pro and antiinflammatory cytokines and endotoxins, which have been targeted in recent decades. Extracorporeal blood purification (EBP) therapies, which use hemoperfusion techniques, have been proposed as adjunctive options to remove excess cytokines and endotoxins, highlighting the importance of innovative approaches in managing sepsis [4].

Various techniques, filters, and membranes have been evaluated for EBP, including CytoSorb hemadsorption, Polymyxin B hemoperfusion, combined endotoxin and cytokine removal by oXiris membranes, Seraph 100 Microbind affinity blood filters, Hemopurifier, and Fc-mannose-binding lectin [5–7]. In recent decades, the clinical usefulness and safety of a resin-based adsorbent cartridge known as HA330 (Jafron Biomedical Co. Ltd., Zhuhai, China) has also been demonstrated for its ability to eliminate cytokines [8–10]. The HA330 cartridge has a pore size of 500 Da to 60 kDa and has been shown to remove molecules ranging from 10–60 kDa, including cytokines and complement factors, under cytokine storm conditions, such as sepsis, resulting in improved 28-day survival [11].

There are currently insufficient strong or convincing data to support the clinical application of EBP therapy, and further attention is warranted. In order to contribute to existing knowledge about purification treatments in septic shock, this study retrospectively evaluated the medical records of patients with septic shock admitted to our intensive care unit (ICU). It aimed to assess the 28-day survival benefit of EBP therapy using the HA330 hemadsorption cartridge compared to conventional therapies.

## 2. Materials and methods

### 2.1. Study design and settings

This study was conducted in a 20-bed capacity medical/surgical mixed-type ICU at a tertiary referral center. The study sample was collected retrospectively from the medical records of patients admitted to the ICU between January 1st, 2015, and July 31st, 2020. It included patients aged ≥18 years with an ICU length of stay (LOS) >96 h (to provide a comparable treatment efficiency of EBP) and diagnosed with septic shock. The definitions of septic shock and its conventional treatment were based on the 2016 guidelines of the European Society of Intensive Care Medicine and the Society of Critical Care Medicine [1]. Patients lost to follow-up within 28 days or readmitted to the ICU were excluded.

### 2.2. Matching process

The case group comprised patients who received EBP therapy in addition to conventional therapies, while the control group comprised patients who only received conventional therapies without EBP. In order to ensure the best possible resemblance and comparability between the 2 groups, the patients were matched 1:1 with controls based on their Acute Physiologic and Chronic Health Evaluation II (APACHE II) scores, which were recorded within the first 24 h of ICU admission or septic shock diagnosis, as appropriate, for the primary analyses.

For the secondary analyses, the patients in the EBP therapy group were assessed for clinical and laboratory improvements 72 h after initiation of the EBP therapy (t = 72).

### 2.3. Data collection

**Figure 1:**
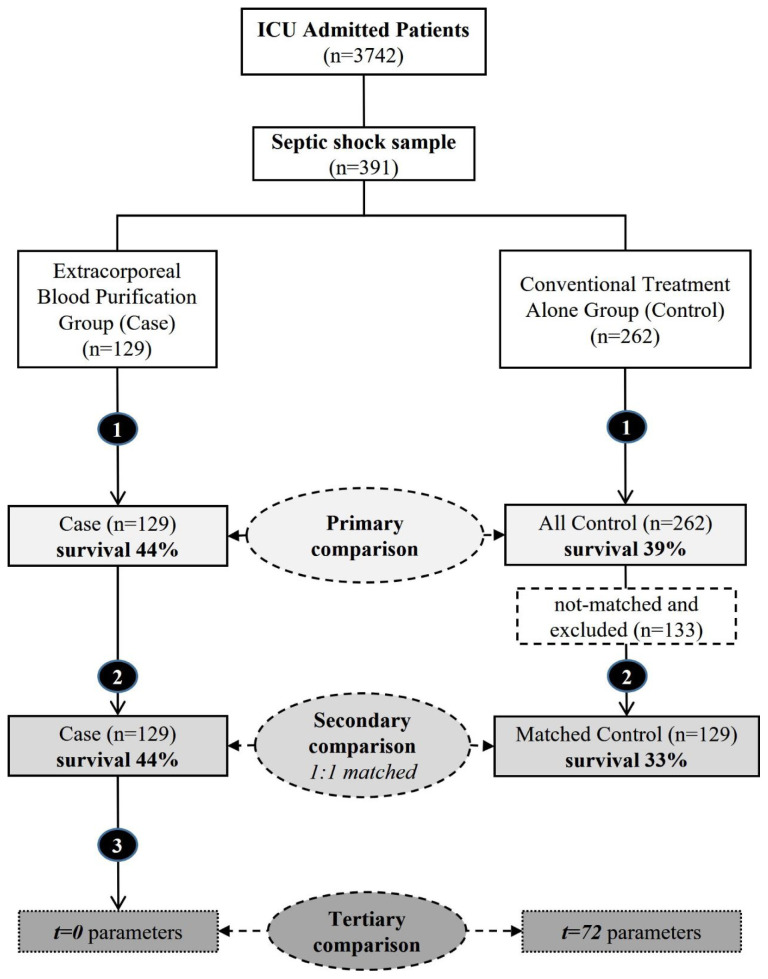
Flow-chart showing the study design, matching, comparisons, and survival rates for the EBP and conventional treatment groups. Unmatched and matched comparisons of the groups, and the tertiary was on parameters recorded at the initiation (t = 0) of EBP therapy and at 72 h (t = 72) after.

The hospital database software and medical record files were used to search and collect the patient data. The following patient information was recorded: sex, age, APACHE II score, and the predicted mortality rate (PMR) calculated using the APACHE II score in the first 24 h. Additionally, the Sequential Organ Failure Assessment (SOFA) score, Shock index (SI), mean arterial pressure (MAP; in mmHg), partial pressure of oxygen in arterial blood (PaO_2_) to the fraction of inspiratory oxygen concentration (FiO_2_; P/F) ratio, peak norepinephrine equivalent (NEE) requirement within 24 h, arterial pH, lactate in mmol/L, C-reactive protein (CRP) level (in mg/L), hematocrit (Hct) level, and platelet (PLT) count (in 10^3^/mm^3^) were also recorded. These parameters were collected at septic shock diagnosis (t = 0) for all of the sampled patients and at 72 h after initiation of the EBP therapy (t = 72) for the EBP group to assess its effectiveness. The parameter values recorded within ±2 h of time points t = 0 and t = 72 were included to ensure reliability and comparability. A diagram illustrating the patient selection process, group allocation, matching procedures, and comparisons is shown in Figure 1.

The NEE variable was calculated by adding the peak doses of norepinephrine, epinephrine, dopamine, and dobutamine (mcg/kg/min) administered within 24 h. Due to its skewed distribution, it was converted and reported as the log_10_NEE. The SI was defined as the heart rate (beats/min) divided by the systolic blood pressure (mmHg).

For the primary comparisons, the 28-day survival status of the patients was recorded. Patient survival was defined as 28-day in-hospital (ICU or ward) survival or home discharge before 28 days. The observed mortality rate (OMR) and PMR were then used to calculate the standard mortality rate (SMR).

Software concealment blinded the data operators during the data search, collection, allocation, matching process, and outcome assessment.

### 2.4. EBP procedure

When performing EBP therapy, the HA330 hemadsorption cartridge was used with the Multi-Filtrate Continuous Renal Replacement Therapy (CRRT) system (Fresenius Medical Care, Bad Homburg, Germany). This system includes a veno-venous dialysis catheter with a serially connected cartridge that allows for venous blood from the patient to enter at one port and be reinfused from the other. A double-lumen catheter percutaneously inserted into the internal jugular or femoral vein provided vascular access. In order to prevent clotting throughout the procedure, an unfractionated heparin (UFH) continuous infusion protocol was used according to activated partial thromboplastin time readings. EBP was conducted daily for at least 4 h for 3 consecutive days, either with or without renal replacement therapies, as necessary.

According to the study clinic’s instructions, EBP was indicated and used in patients with persistent septic shock despite complete recommended attempts for 24 h, and at least 2 of the following criteria were present: a) peak log_10_NEE >2.5, b) SI >1.0, or c) lactate >4 mmol/L. EBP was contraindicated or not used in those with an APACHE II score <20, aged >90 years, unable to be cannulated, unable to undergo systemic anticoagulation, whose hemodynamic status could not be managed with vasopressor/inotropic support for extracorporeal circulation, or with an irreversible underlying disease and when the EBP device or cartridge was unavailable or inaccessible.

### 2.5. Statistical analyses

Continuous variables were reported as the mean ± standard deviation (SD), while the categorical variables were presented as frequencies (n) and percentages (%). Parametric data, repeated measures, and survival rates were compared using t-tests, and survival functions were compared using Kaplan–Meier log-rank analyses. The data were weighted using the patients’ APACHE-II scores. Odds ratios (ORs), absolute risk reductions (ARR), relative risk reductions (RRRs), number needed to treat (NNT), sensitivity, and specificity were calculated using contingency tables. The analyses were 2-tailed, and p < 0.05 was considered statistically significant. Data management and analyses were performed using IBM SPSS Statistics for Windows 25.0 (IBM Corp., Armonk, NY, USA).

### 2.6. Study aim

The primary aim of this study was to evaluate the 28-day survival benefit of EBP therapy using the HA330 hemadsorption cartridge compared to conventional treatments. Its secondary aim was to assess the effect of EBP therapy on selected parameters.

### 2.7. Ethical aspects

This study was ethically approved by the Bioethical Board of Trakya University (no.2020/252–12/03). It was conducted according to the 2008 Declaration of Helsinki. Written informed consent was obtained from the parent/legal guardian/next of kin of the participants to participate in this study. Per the study clinic’s regulatory procedures, patients or their legally authorized relatives provided written informed consent for processing and publishing patients’ medical records (with names disclosed) for scientific purposes.

## 3. Results

This study included 391 of 3742 patients admitted during the defined period. Among them, 129 patients with septic shock received EBP therapy (EBP group), accounting for 33% of the patients. The average initiation time for EBP therapy was 28 (25–38) h. The control group comprised 262 patients who received conventional therapy without EBP. Table 1 presents the participants’ sex, age, APACHE II scores, and laboratory parameters at initiation to the study in the EBP and control groups (t = 0). The 28-day survival rate was higher among patients in the EBP group (44%) than in the control group (39%, p < 0.05) despite the fact that they had higher calculated APACHE II scores, SIs, peak log_10_NEEs, and lactate levels, which predict a worse prognosis.

**Figure 2:**
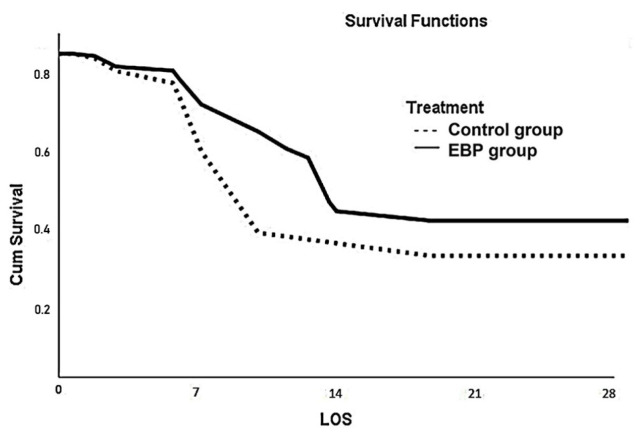
The 28-day Kaplan–Meier survival graph of the EBP and conventional treatment control groups.

A best 1:1 matching was performed, excluding 133 unmatched patients, and leaving 129 patients in both the EBP and control groups. The calculated PMR was higher for the EBP group (67%) than the matched control group (60%). However, the matched control group had a significantly lower 28-day survival rate (n = 43, 33%) than the EBP group (n = 57, 44%; p = 0.001, log-rank = 0.05), indicating a better SMR. The Kaplan–Meier survival curve for the 28-day period is shown in Figure 2. The beneficial effects of EBP therapy were demonstrated by favorable 37% RRR and 12% ARR rates (OR = 1.7, NNT = 8), although with moderate sensitivity (57%) and specificity (56%). The results are shown in Table 2.

Clinical and laboratory parameters were compared at the initiation (t = 0) of EBP therapy and at 72 h (t = 72) after. EBP therapy significantly improved the patients’ SOFA scores, SIs, P/F ratios, peak log_10_NEEs, pH, lactate levels, and CRP levels after 72 h of therapy (p < 0.05). The most significant improvements were in the P/F ratios, NEEs, and lactate levels (p < 0.001). These results are shown in Table 3.

However, the mean MAP level did not change, possibly due to NEE dose adjustments required to stabilize the patients’ clinical conditions. In addition, the Hct levels did not change, while the PLT levels decreased. No transfusion requirements or bleeding complications were recorded.

## 4. Discussion

EBP therapies have gained attention in recent years due to promising results published in treating patients with septic shock [4,12,13]. Different filters and membranes have been developed and successfully used in recent decades, with resin cartridges among the feasible and beneficial options [5–10].

This retrospective study investigated EBP therapy with resin HA330 cartridges, finding that it significantly improved 28-day survival in patients with septic shock compared to conventional treatment alone. However, the calculated sensitivity, specificity, and ORs were only moderate in the matched analyses.

Brouwer et al. compared PMRs versus OMRs in patients with septic shock, demonstrating that EBP had significant survival benefits [5], consistent with the current findings. The study herein also showed that EBP therapy significantly improved the NEE dose requirements and facilitated lactate clearance, similar to the studies by Friesecke and Hawchar [14–16]. Two studies by Huang et al. showed that EBP with HA330 resin cartridges significantly decreased the vasopressor requirements of patients with septic shock [8,9], consistent with the current findings. In addition, the present study demonstrated a significant improvement in the patients’ SIs, which are related to their survival. Higher SIs have been considered mortality predictors for ICU patients and a trigger for aggressive treatment approaches [17]. The P/F ratios of the EBP patients herein were also significantly improved after 72 h of therapy, consistent with some previous resin cartridge studies [8,9,18]. Studies by Huang et al. found that improved P/F ratios were correlated with cytokine clearance and better ICU survival rates [8,9]. In the current study, the CRP levels, an indirect indicator of cytokine status, were significantly decreased. Recently, Kaçar et al. reported that EBP therapy with HA330 cartridges significantly improved CRP levels in their patients with septic shock, although other parameters and prognosis were unchanged [19]. It was assumed that the survival difference between the current study and theirs was due to the different study setups, patient populations, and timing of the EBP initiation.

Another issue was when to initiate the EBP therapy. It has been suggested that earlier initiation would result in better survival [20–22]. Some studies have assessed EBP therapy as only a rescue option, and its initiation was delayed. They found that the survival benefit was negligible [14,20–22], likely because the patient’s clinical condition had already deteriorated almost beyond recovery. In the present study, the initiation of EBP was standardized at 24 h of persistent septic shock status despite complete supportive measures, as defined by our clinic guidelines, which were similar to the recommendations of the ACCESS trial [14,22].

The length of time a filter maintains an effective adsorptive capacity has been a controversial issue in EBP therapies. Its capacity decreases with time due to saturation, and the elimination of cytokines is negated by the shift from the interstitial to the blood compartment, which could affect the survival benefit. Some studies have recommended that filter usage should not exceed 4 h [23–25]. It was also our opinion herein that HA330 cartridges should not be used for more than 4 h for optimum benefit; thus, a new cartridge was used each day, as recommended by the manufacturer. This issue should be considered when assessing the conflicting results from different studies.

Some studies have encouraged using APACHE II or SOFA scores in the decision to initiate EBP therapy for further benefit [14,16]. We agree that this would be an important addition to standardized EBP approaches. Since generally accepted indications have not been precisely defined, EBP therapy has been applied in different indications and conditions in many studies, weakening the generalizability of their results. The current study considered an APACHE II score of <20 as a relative contraindication.

The lack of established guidelines for EBP therapies has led to variability in clinical practices. EBP therapies have been initiated based on the judgement of the responsible physicians and local practice and expertise levels. A recent web-based, multicenter, observational prospective registry, Extracorporeal Blood Purification Therapy in Critically Ill Patients (GlobalARRT), aims to enroll 1000 participants and is expected to be completed in 2023 (ClinicalTrials.gov identifier: NCT04580680). This registry is not yet recruiting and was last updated on October 9th, 2020, and last visited on October 23rd, 2020. This registry defines short-term outcomes as a ≥20% decrease in vasoactive inotropic needs, improvement in hemodynamic stability and inflammatory status, and improvement in clinical parameters at 12, 24, and 48 h after EBP initiation. Long-term outcomes were defined as patient survival 10 days after EBP initiation and ICU discharge. These aspects of the registry design are similar to those herein. This registry aims to explore initiation timing, clinical circumstances, clinical variables, utilization rates, technical characteristics, chosen anticoagulation strategies, and average flow rates used for EBP therapies in ICUs worldwide. Its results would provide a wide range of data and clarify most conflicting issues on EBP therapies.

### 4.1. Limitations

Due to its retrospective design, this study was susceptible to historical sampling, selection, exclusion, recall, or attrition biases, and there may have been unrecognized confounders. In addition, not all of the parameters or characteristic measures were included in the statistical analyses for the outcome, even though some were already accounted for in the SOFA or APACHE II scores. Multivariate regression analyses could have been conducted using all possible measures and parameters. Cytokine levels were not measured but CRP levels were used as an indirect parameter. Care was also made to avoid comparing different cartridges with different compositions and capabilities, instead focusing on the efficiency of EBP therapy in our clinic and similar studies using HA330 cartridges.

## 5. Conclusion

EBP studies conducted over the past 2 decades have reported conflicting results, likely due to variability in the patient populations, disease severity, EBP indications, cartridges, procedures, initiation times, and therapy durations across different studies. However, we propose that improvements in the NEEs, lactate levels, SIs, CRP levels, and P/F ratios all contributed to the improved septic shock status and, ultimately, the survival of the patients included herein, as reflected in their lower SOFA scores within 72 h of EBP therapy initiation. While the calculated ORs were moderate, the estimated NNT was promising. We suggest that EBP could be a promising and beneficial therapy option for select patients with septic shock, but standardization of the indications and procedures is necessary. We call for future randomized controlled trials with larger sample sizes to further evaluate the feasibility and efficacy of EBP therapies.

## Figures and Tables

**Table 1:** Presentation of sex, age, and Acute Physiologic and Chronic Health Evaluation II (APACHE II) scores, clinical, and laboratory parameters, and overall survival rates for the EBP and unmatched control groups, at initiation t = 0.

	EBP group	Unmatched control group	p-value
Total, n (female, n)	129 (49)	262 (92)	
Age (years)	62 ± 16	67 ± 9	ns
APACHE II score	22 ± 7	19 ± 8	<0.05
SOFA score	13 ± 1	10 ± 3	ns
SI	1.04 ± 0.95	1.02 ± 0.54	<0.01
MAP (mmHg)	74 ± 11	68 ± 9	ns
P/F ratio	252 ± 106	273 ± 56	ns
peak log_10_NEE	2.5 ± 0.6	2.2 ± 0.4	<0.01
pH	7.359 ± 0.106	7.339 ± 0.121	ns
Lactate (mmol/L)	2.6 ± 1.1	2.3 ± 1.2	<0.05
CRP (mg/L)	22 ± 11	22 ± 8	ns
Hct	29 ± 5	30 ± 4	ns
PLT (10^3^/mm^3^)	112 ± 101	105 ± 42	<0.05
Survival n (%)	57 (44%)	103 (39%)	<0.05

SOFA: Sequential Organ Failure Assessment score, SI: shock index, MAP: mean arterial pressure, P/F ratio: PaO2/FiO2 ratio, peak log_10_NEE: peak norepinephrine equivalent requirements in log_10_ within 24 h, CRP: C-reactive protein, Hct: hematocrit, PLT: platelet, ns: statistically nonsignificant.

**Table 2:** Presentation of the observed mortality rates (OMR) and predicted mortality rates (PMR) (using the APACHE II score) and calculated standardized mortality ratios (OMR/PMR) for the EBP and 1:1 matched control groups. Relative risk reduction (RRR), absolute risk reduction (ARR), odds ratio (OR) (given with 95% confidence intervals), and number needed to treat (NNT) values (sensitivity: 57%, specificity: 56%).

	EBP group (n = 129)	Matched control group (n = 129)
Survival	44%* (n = 57)	33% (n = 43)
OMR (n)	56% **	67%
PMR	67% *	60%
SMR	0.84 *	1.12
RRR	37% (29–51)	
ARR	12% (8–7)	
OR	1.7 (1.3–2.1)	
NNT	8	

* statistically significance at p < 0.05,

** statistically significance at p < 0.001.

**Table 3:** Comparison and presentation of the clinical and laboratory parameters recorded at the initiation (t = 0) at the initiation (t = 0) of EBP therapy and at 72 h (t = 72) after.

	t = 0	t = 72	p-value
SOFA	13 ± 1	12 ± 4	<0.05
SI	1.04 ± 0.95	0.93 ± 0.24	<0.05
MAP (mmHg)	74 ± 11	76 ± 14	ns
P/F ratio	252 ± 106	362 ± 131	<0.001
peak log_10_NEE	2.5 ± 0.6	2.1 ± 0.5	<0.001
pH	7.359 ± 0.106	7.393 ± 0.067	<0.05
Lactate (mmol/L)	2.6 ± 1.1	1.9 ± 1.5	<0.001
CRP (mg/L)	22 ± 11	20 ± 6	<0.05
Hct	29 ± 5	28 ± 5	ns
PLT (10^3^/mm^3^)	112 ± 101	96 ± 83	<0.05

ns: Statistically nonsignificant.

## Data Availability

The research data are not publicly available for legal reasons. Please e-mail the corresponding author for data access.
